# Enhanced synaptic plasticity and spatial memory in female but not male FLRT2-haplodeficient mice

**DOI:** 10.1038/s41598-018-22030-4

**Published:** 2018-02-27

**Authors:** Ana Cicvaric, Jiaye Yang, Tanja Bulat, Alice Zambon, Manuel Dominguez-Rodriguez, Rebekka Kühn, Michael G. Sadowicz, Anjana Siwert, Joaquim Egea, Daniela D. Pollak, Thomas Moeslinger, Francisco J. Monje

**Affiliations:** 1 Department of Neurophysiology and Neuropharmacology, Center for Physiology and Pharmacology, Medical University of Vienna, Schwarzspanierstrasse 17, 1090 Vienna, Austria; 2 Molecular and Developmental Neurobiology Research Group, Universitat de Lleida - IRBLleida, Office 1.13, Lab. 1.06. Avda. Rovira Roure, 80, 25198 Lleida, Spain; 3 Institute for Physiology, Center for Physiology and Pharmacology, Medical University of Vienna, Schwarzspanierstrasse 17, 1090 Vienna, Austria

## Abstract

The Fibronectin Leucine-Rich Transmembrane protein 2 (FLRT2) has been implicated in several hormone -and sex-dependent physiological and pathological processes (including chondrogenesis, menarche and breast cancer); is known to regulate developmental synapses formation, and is expressed in the hippocampus, a brain structure central for learning and memory. However, the role of FLRT2 in the adult hippocampus and its relevance in sex-dependent brain functions remains unknown. We here used adult single-allele FLRT2 knockout (FLRT2^+/−^) mice and behavioral, electrophysiological, and molecular/biological assays to examine the effects of FLRT2 haplodeficiency on synaptic plasticity and hippocampus-dependent learning and memory. Female and male FLRT2^+/−^ mice presented morphological features (including body masses, brain shapes/weights, and brain macroscopic cytoarchitectonic organization), indistinguishable from their wild type counterparts. However, *in vivo* examinations unveiled enhanced hippocampus-dependent spatial memory recall in female FLRT2^+/−^ animals, concomitant with augmented hippocampal synaptic plasticity and decreased levels of the glutamate transporter EAAT2 and beta estrogen receptors. In contrast, male FLRT2^+/−^ animals exhibited deficient memory recall and decreased alpha estrogen receptor levels. These observations propose that FLRT2 can regulate memory functions in the adulthood in a sex-specific manner and might thus contribute to further research on the mechanisms linking sexual dimorphism and cognition.

## Introduction

Leucine-Rich Repeat motifs (LRR) are protein domains of approximately 30 amino acids with proportionally high number of Leucines^1,2^. LRR domains play central roles in cellular physiology as they confer proteins with the capability to regulate a variety of vital functions, including protein-protein interactions, ligand binding and cellular adhesion, or repulsion, and cell growth^3^. In the human brain, proteins containing LRR domains are known to regulate neurogenesis, synaptic plasticity, and memory functions^4,5^. Moreover, several LRR proteins have been implicated in Alzheimer’s disease^6–8^, Parkinson’s disease^9–12^ and epilepsy^13,14^.

FLRT2 is a member of the Fibronectin Leucine Rich Repeat Transmembrane (FLRT) family of proteins, which are highly conserved in evolution^15–18^. It has a relatively short intracellular region (~14% of the protein length), a single type-III Fibronectin-like domain (~8% of protein length) and, most distinctively, ten extracellular LRR domains comprising (in the human) approximately 60% of the entire protein^16,19,20^. During embryological development, FLRT2 has been localized in the mammalian brain^21,22^ and has been implicated in neuronal migration, synapse formation, and brain neural circuit formation^21,22^. Interestingly, FLRT2 can be also found in the adult brain, with marked expression in the hippocampus^23^, suggesting an involvement in hippocampal neuronal functions. However, the role *in vivo* of FLRT2 in synaptic activity and hippocampus-dependent learning and memory remains, to the best of our knowledge, completely unknown.

Here, we used FLRT2 heterozygous knockout mouse (FLRT2^+/−^) as experimental animal model to investigate the role of FLRT2 on adult hippocampal synaptic plasticity and hippocampus-dependent learning and memory functions. Conventional full knockouts animals were not used in the present study as previous reports demonstrated that the vast majority of homozygous FLRT2 knockout animals die at embryonic day 12–15^18,22^ and therefore cannot be used to study the importance of FLRT2 in postnatal brain functions. The use of heterozygous knockout animals constitute a widely accepted tool for the identification of molecular components implicated in the regulation of synaptic plasticity and higher order cognitive functions^24–26^. Our observations of the effects of the genetic suppression of FLRT2 via haplodeficiency in female and male mice propose FLRT2 as a critical molecular component regulating adult synaptic plasticity and suggest a sex-dependent implication of FLRT2 in the promotion of hippocampus-dependent memory functions.

## Results

### Wild type Vs FLRT2^+/−^ mice major physical traits

**Figure 1:**
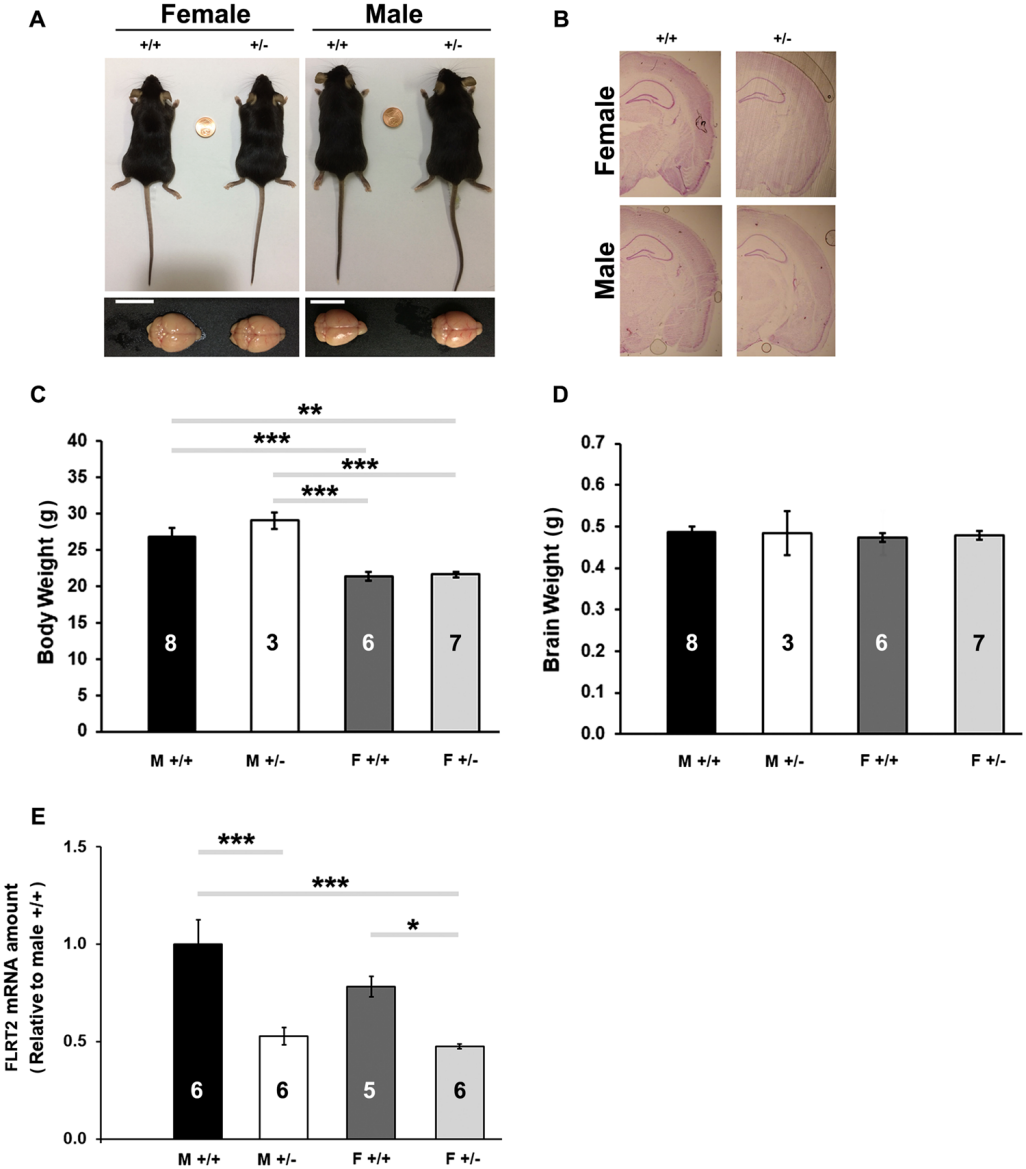
FLRT2+/− mice do not present with gross, detectable phenotypical abnormalities. (**A**) Adult male or female FLRT2^+/−^ mice do not present with distinctive gross morphological abnormalities in body or brain structure. (**B**) Microphotographs of Nissl-stained coronal brain sections showed no obvious differences in shape and major cytoarchitectonic brain organization between wild type and FLRT2^+/−^ male or female mice. (**C**) Significant main effect of the sex was observed for body weights (*p* < 0.001, *F*_(1,20)_ = 44.07) with a consistent pattern between wild type and FLRT2^+/−^ mice. (**D**) No significant differences were observed in brain weights (ns, p > 0.05). (**E**) FLRT2^+/−^ males and females presented with significantly lower levels of FLRT2 mRNA in the hippocampus as compared to their respective wild type littermate controls, thus exhibiting a main effect of the genotype (*p* < 0.0001, F_(1,19)_ = 27.93). Results of the *post hoc* test are indicated in the graph by asterisks. *p ≤ 0.05; **p ≤ 0.01; ***p ≤ 0.001; ****p ≤ 0.0001. Number of subjects is indicated within bar charts. Data are displayed as mean ± SEM, sample sizes are indicated inside the bars.

Previous reports have shown that some members of the FLRT family of proteins can function as regulators of early embryonic vascular and neural development in mice^18,21,27–30^. Specifically, FLRT2 has been shown to be important for heart morphogenesis, maintaining integrity of the epicardium tissue and in the organization of the basement membrane architecture^18^. Consistent with this function, FLRT2 deficiency causes lethality to the vast majority of embryos at mid-gestation due to cardiac insufficiency^18^. Additionally, selective FLRT2 knock down in the endothelial cells has been linked to the aberrant placental labyrinth formation and full mid-gestation lethality^29^. However, others^18^ and we here observed that heterozygous FLRT2^+/−^ offspring appear healthy and fertile as adults. Therefore, we here used FLRT2^+/−^ mice as an animal model to investigate the role of FLRT2 proteins on the brain function. To this aim, we examined several basic phenotypical factors in order to exclude possible abnormalities that could emerge from FLRT2 haplodeficiency throughout development and pose confounding factors in our study. First, we examined the general morphology of the body and brain and found that both male and female mice carrying a single allele FLRT2 mutation were indistinguishable in their appearance from their sex-respective wild type (FLRT2^+/+^) littermates (Fig. 1A). Moreover, basic histological examination of the Nissl stainings did not reveal any detectable neuroarchitectural abnormalities in the structure and the layering of the major areas of the adult brain of FLRT2^+/−^ mice (Fig. 1B). When body weights were examined, wild type males had weights greater than those of females, as it is characteristic for this specie, a pattern also present in FLRT2^+/−^ mice. No significant differences in body (Fig. 1C) and brain (Fig. 1D) weights were observed for either male or female FLRT2^+/−^ mice when compared to their sex-respective wild type littermate controls. qRT-PCR of hippocampal tissue showed that the single FLRT2 allele deletion results in a reduction of the FLRT2 mRNA levels in both in male (~47% reduction) and in female (~40% reduction) mice (Fig. 1E), which is in agreement with the inhibition in the level of expression of specific proteins in other studied single-allele knockout mice^24–26^.

### Behavioral analysis of FLRT2^+/−^ mice shows sex-specific changes in memory recall

**Figure 2:**
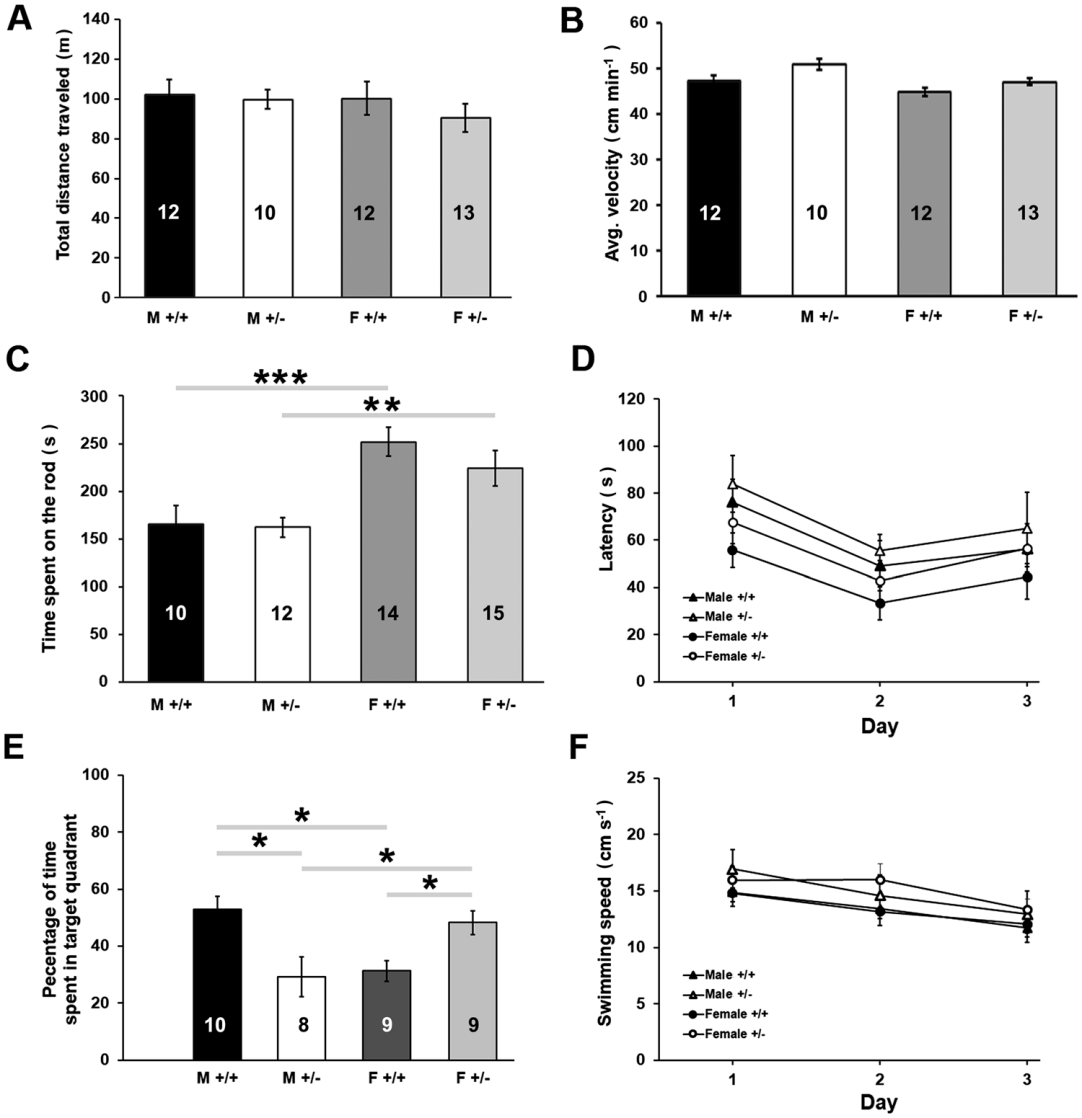
Behavioral analysis of FLRT2^+/−^ mice shows sex-specific changes in memory recall. Open field test did not reveal significant changes in locomotor activity in male or female FLRT2^+/−^ mice compared to their corresponding wild type littermates, as determined by measuring (**A**) total distance traveled. When the average velocity during the test was examined, however, a significant main effect of sex (*p* = 0.004, *F*_(1,46)_ = 9.23) and a main effect of genotype (*p* = 0.009, *F*_(1,46)_ = 7.72) was apparent as indicated in (**B**) with data showing no significance differences between groups after *post hoc* analysis. (**C**) Examinations in the accelerating Rota-rod test showed a significant main effect of sex (*p* < 0.00001, *F*_(1,50)_ = 20.01) with no main effect of genotype on motor coordination. (**D**) No significant differences in spatial learning were detected between either male or female FLRT2^+/−^ mice when compared to the their respective wild type littermate controls as determined by escape latencies during training days in the Morris water maze (*p* > 0.05, n = 8–14). However, there was a statistically significant interaction between the effects of sex and genotype on long-term memory retention (**E**) during probe trial (*p* = 0.0001, *F*_(1,32)_ = 19.43, n = 8–14). (**F**) No significant differences in swimming speeds were observed (p > 0.5, n = 8–14). Latencies to reach the hidden platform and average swimming speeds displayed in the graph are averages of three trials per day. Results of the post hoc test are indicated in the graph by asterisks. *p ≤ 0.05; **p ≤ 0.01; ***p ≤ 0.001; ****p ≤ 0.0001. Data are displayed as mean ± SEM; sample sizes are indicated inside the bars.

Before examining the effects of single-allele FLRT2 deletion on hippocampus-dependent learning and memory functions, we examined the impact of this mutation on locomotor activity by comparing the performance of adult male and female FLRT2^+/−^ mice to their sex-corresponding wild type littermate controls in the open field test (OFT). No significant main effect of sex or genotype, nor sex x genotype interactions in total distance traveled (Fig. 2A) were observed indicating that having lower levels of FLRT2^+/−^ does not interfere with regular spontaneous locomotor activity. When average velocity during the test was measured (Fig. 2B), significant main effect of the sex and main effect of the genotype were observed with no significant interaction between the two factors. However, *post hoc* analysis showed no significant differences between groups. Additionally, motor coordination was examined by assessing the performances in the accelerating Rota-rod test of adult male and female mice. Compatible with observations from other authors^31^, a difference in performance was observed between male and female wild type animals. This pattern was also observed when male vs female FLRT2^+/−^ mice were compared, showing a significant main effect of sex on motor coordination (Fig. 2C). No significant differences were found between male wild type vs male FLRT2^+/−^ mice or between female wild type vs female FLRT2^+/−^ mice (Fig. 2C). We next examined the impact of the single-allele FLRT2 deletion on learning and memory employing the Morris Water Maze test (MWM), which is a standardized test widely used to examine the properties of hippocampus-dependent spatial learning and memory^32,33^. During all training days, there were no observable differences in the escape latencies of either male or female heterozygous FLRT2^+/−^ mice when compared to those of their corresponding wild type littermate controls (Fig. 2D). Taken together, these observations indicate that the FLRT2 single-allele deletion does not results in any deleterious impact either on gross motor performance or on the initial phases of learning acquisition. We additionally conducted a probe trial in order to examine spatial memory retention in heterozygous FLRT2^+/−^ mice. A two-way ANOVA revealed a sex x genotype interaction with male FLRT2^+/−^ mice spending less and female FLRT2^+/−^ spending more time in the target quadrant where the platform was placed during the training period compared to their respective wild-type littermates (Fig. 2E). These observations suggest a sex-dependent effect of FLRT2 haplodeficiency on memory recall. Examinations of swimming speeds during the performance in the water maze, in both male and female FLRT2^+/−^ mice, showed that both male and female heterozygous FLRT2^+/−^ mice presented with swimming speeds comparable to those of their sex-respective littermate wild type controls (Fig. 2F). These observations propose that FLRT2 might participate *in vivo* in hippocampus-mediated memory recall in a sex-specific manner.

### Loss of a single FLRT2 allele enhances hippocampal synaptic plasticity in female but not male mice

**Figure 3:**
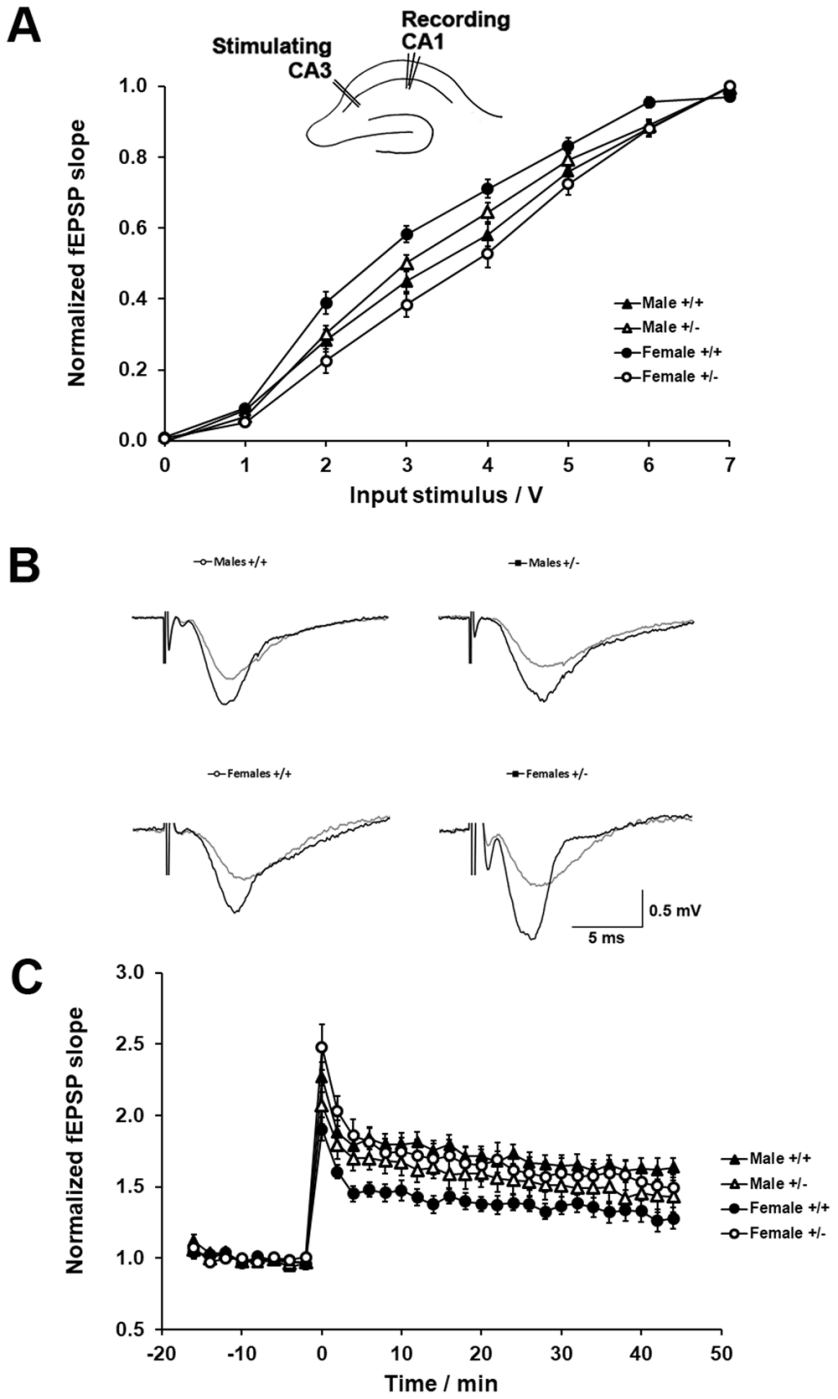
Loss of a single FLRT2 allele alters synaptic plasticity in female but not male mice. (**A**) Analysis of basal synaptic transmission (input/output curves) in CA3-Schaffer collateral-CA1 synapses. Main significant effect of voltage (*p* < 0.0001, *F*_(7, 385)_ = 1885, n = 11–18), groups (p = 0.002, *F*_(3, 55)_ = 6.0, n = 11–18) and interaction (*p* < 0.0001, *F*_(21, 385)_ = 4.8) were observed. *Post hoc* analysis showed significant difference between male and female wild type mice (*p* = 0.02, n = 11–18) and between female wild type and female FLRT2^+/−^ mice (*p* = 0.002, n = 11–18). Inset cartoon on top represents the approximate positioning of the stimulating and recording electrode. (**B**) Representative traces of field excitatory postsynaptic potential recorded from animals from respective sex and genotype as indicated. (**C**) Temporal courses of averaged slopes of fEPSP showed a main significant effect of time (*p* < 0.0001, *F*_(90, 4140)_ = 32.6, n = 11–13), a significant main effect between groups (*p* = 0.03, *F*_(3, 46)_ = 3.6, n = 11–13) and a significant interaction (*p* < 0.0001, *F*_(270, 4140)_ = 1.6). *Post hoc* analysis revealed statistically significant differences between male and female wild type mice (*p* = 0.03, n = 11–13) and between female wild type and female FLRT2^+/−^ mice (*p* = 0.04, n = 11–13). *p ≤ 0.05; **p ≤ 0.01; ***p ≤ 0.001; ****p ≤ 0.0001. Data are displayed as mean ± SEM.

To examine the potential implication of FLRT2 in the hippocampal synaptic function, we examined *ex vivo* the electrophysiological properties of the monosynaptic circuit established between the CA3 region and the pyramidal inputs of the CA1 region via the Schaffer collateral pathway, a circuit proposed to be implicated in the formation and maintenance of spatial memories^34^. To this aim, we first analyzed the properties of basal synaptic transmission using standard input/output protocols (Materials and Methods) in hippocampal slices obtained from male or female wild type controls and FLRT2^+/−^ mice. No differences in the voltage-dependence of synaptic transmission were detected between male FLRT2^+/−^ and their corresponding wild type controls and a right shift was observed in FLRT2^+/−^ female mice as compared to their related wild type female controls (Fig. 3A). Next, we examined the properties of long-term synaptic potentiation (LTP) in hippocampal slices from wild type controls and heterozygous FLRT2^+/−^ male and female mice. Recordings of extracellular field potentials and analysis of field-slopes indicated that whereas no significant differences were apparent between male FLRT2^+/−^ and male wild type mice, a significant enhancement in synaptic potentiation was observed in female FLRT2^+/−^ mice relative to their wild type female littermates (Fig. 3B,C).

### Female but not male FLRT2+/− mice have reduced levels of EAAT2

**Figure 4:**
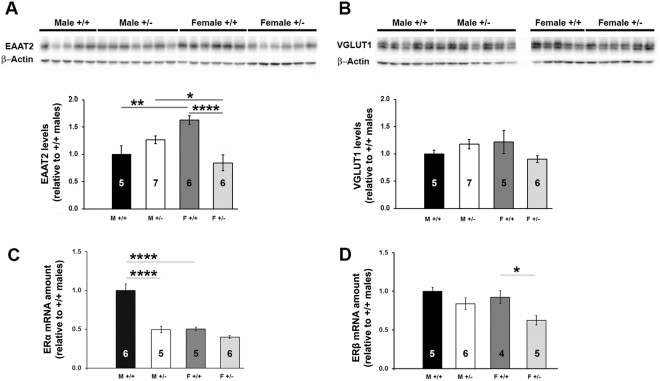
FLRT2 haplodeficiency results in reduced hippocampal levels of EAAT2 and ERβ only in females. (**A**) Representative pictures of western blot assays (upper panel) and bar charts (below) for densitometric analyses. A significant main effect of the genotype on the protein levels of EAAT2 (*p* = 0.02, *F*_(1, 20)_ = 7.394, n = 5–7) and a significant interaction between effects of the genotype and sex (*p* < 0.0001, *F*_(1, 20)_ = 30.94) was observed. Blot images were cropped for comparisons; full-length blots are available in the supplementary data. (**B**) No differences between groups were observed for VGLUT1 levels (*p* > 0.5, n = 5–7). Blot images were cropped for comparisons; full-length blots are available in the supplementary data. (**C**) A strong effect of sex (*p* < 0.0001, *F*_(1, 17)_ = 31.98, n = 6) and a strong main effect of the genotype (*p* < 0.0001, *F*_(1, 17)_ = 33.32, n = 6) on the hippocampal ERα mRNA levels, as well as a significant interaction between two factors (*p* = 0.003, *F*_(1, 17)_ = 12.8, n = 6) were observed. (**D**) qRT-PCR revealed only a significant effect of genotype on the ERβ mRNA levels (p = 0.004, *F*_(1, 16)_ = 11.32, n = 6). Results of the post hoc test are indicated in the graph by asterisks. *p ≤ 0.05; **p ≤ 0.01; ***p ≤ 0.001; ****p ≤ 0.0001. Data are displayed as mean ± SEM; sample sizes are indicated inside the bars.

Glutamatergic signaling plays a key role in the modulation of hippocampal synaptic plasticity and learning and memory^35^. Previous studies have also shown that 17β-estradiol (E2), the primary female sex hormone, can up-regulate both the mRNA and protein levels of the excitatory amino acid transporter 2 (EAAT2, known as GLT-1 in rodent literature)^36,37^. Therefore, in an effort to address potential molecular signaling pathways that could underlie the sex-dependent memory phenotype observed in FLRT2^+/−^ mice, we examined the levels of the vesicular glutamate transporter 1 (VGLUT1) and of EAAT2 in FLRT2^+/−^ mice by conventional Western blot assay. Whereas no changes were observed for the levels of VGLUT1 in either male or female mice, FLRT2^+/−^ females exhibited significantly reduced levels of EAAT2 compared to their female counterpart, while no differences were observed between wild type and FLRT2^+/−^ males (Fig. 4A,B).

### Sexually dimorphic levels of ERα and ERβ in FLRT2 haplodeficient mice

Estrogen receptor-mediated signaling plays a central role in the regulation of the adult brain function and has a critical impact on neuronal morphology, synaptic plasticity and learning and memory processes^38–45^. Given the sex-dependent differences in behavior and synaptic plasticity observed upon FLRT2 haplodeficiency, we also examined the levels of the Estrogen Receptor Alpha (ERα) and the Estrogen Receptor Beta (ERβ) in male and female FLRT2^+/−^ mice hippocampi. Interestingly, while the levels of ERα were dramatically reduced only in male FLRT2^+/−^ mice (Fig. 4C), the levels of ERβ were significantly reduced only in female FLRT2^+/−^ mice compared to those of their corresponding littermate controls (Fig. 4D), suggesting that FLRT2 haplodeficiency has a differential impact on the levels of estrogen receptors depending on the sex.

### Female FLRT2 haplodeficient mice exhibit altered hippocampal CA1 pyramidal neurons apical dendritic complexity

**Figure 5:**
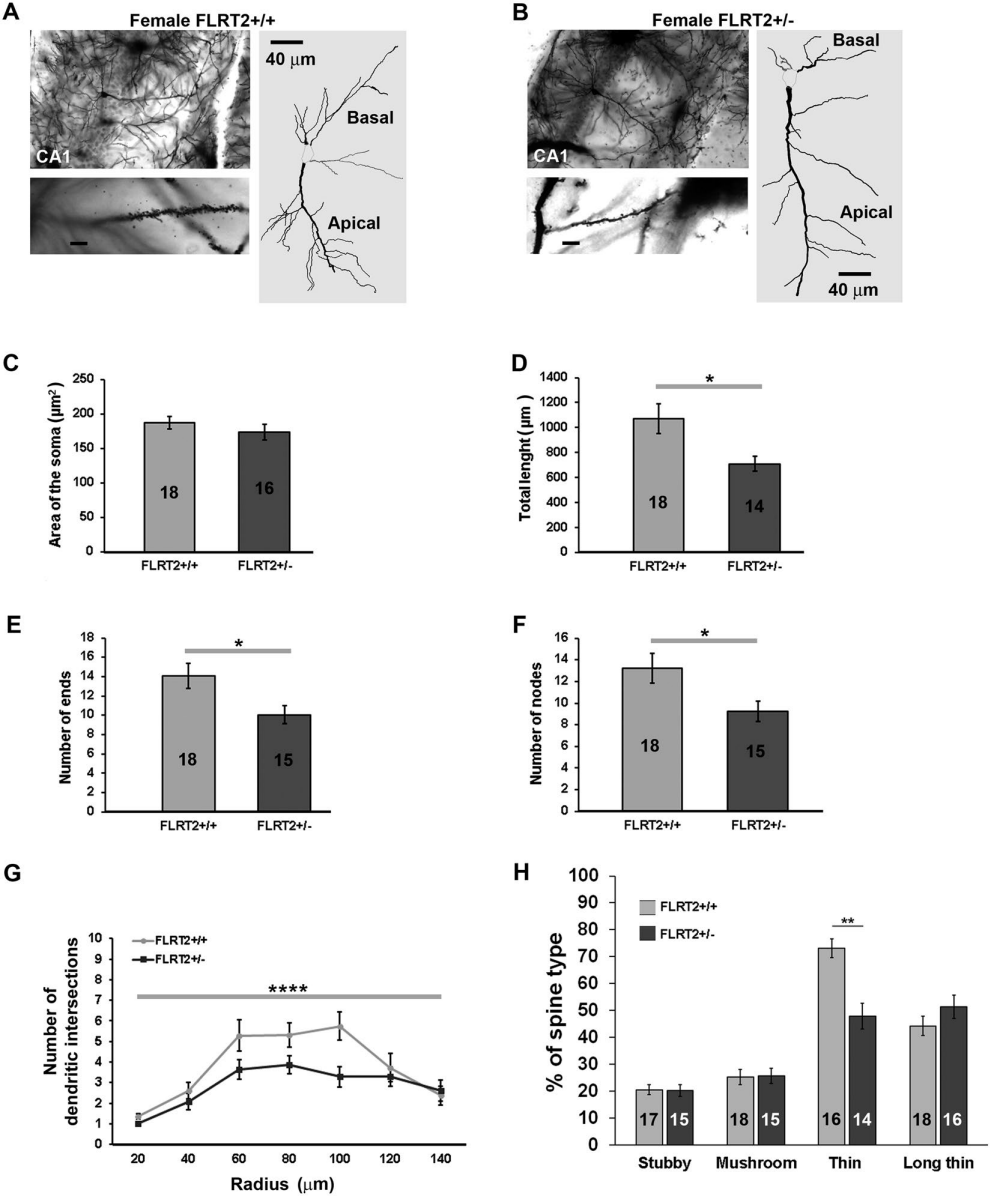
FLRT2 haplodeficiency affects apical dendritic complexity in hippocampal CA1 pyramidal neurons of female mice. Representative photomicrographs of brain sections showing dendritic arborizations of neurons from female FLRT2^+/+^ (**A**), upper left panel and FLRT2^+/−^ mice (**B**), upper left panel as visualized by Golgi-Cox staining. Representative Neurolucida digital neuronal reconstructions are shown in the right panels (in **A**,**B**). Higher magnification (100 × oil objective) photomicrographs of tertiary apical branches are also depicted (lower left panels in A and B; Scale bar = 5.8 µm). (**C**–**F**) Quantification of the area of hippocampal CA1 pyramidal neuronal somata; total length of apical dendritic tree and number of ends and number of nodes, respectively. (**G**) Sholl analyses of the total number of dendritic intersections within each shell (as examined using a two-way ANOVA) showed a significant effect of the genotype (p < 0.0001) and a significant effect of radius (p < 0.0001) on the number of intersections. (**H**) Quantification Stubby, Mushroom, Thin and Long thin dendritic spines in CA1 pyramidal neurons from female FLRT2^+/+^ and FLRT2^+/−^ mice. Data are represented as mean ± SEM; sample sizes are indicated inside the bars. *p ≤ 0.05; **p ≤ 0.01; ***p ≤ 0.001; ****p ≤ 0.0001.

The morphological characteristics of neurites and dendritic spines (e.g. neuritic length, spine number and morphology) in hippocampal neurons play key roles in determining synaptic activity and memory-related plasticity functions^46,47^. Moreover, FLRT3, a member of the FLRT family of proteins also expressed in the hippocampus, has been implicated in the regulation of neurite outgrowth and synaptic development^20^. On these grounds, and given that our observations indicate an involvement of FLRT2 in hippocampal synaptic plasticity, we next explored whether the functional effects of FLRT2 haplodeficiency could be accompanied by morphological alterations in dendritic complexity. To this aim, we conducted Golgi-Cox staining of hippocampal CA1 pyramidal neurons obtained from wild type and FLRT2^+/−^ female mice (materials and Methods). Since our data indicated a possible sex-hormone-related activity of FLRT2, and as the effect of estradiol (perhaps the most important sex-related hormone in mice) on dendritic spines (of hippocampal CA1 pyramidal cells) is more pronounced on the apical tree^46^ (see also^48^), we therefore focused our analyses on this neuronal structure (Fig. 5). Whereas no significant differences in general, macroscopic appearance or in the area of the soma were detected (Fig. 5A–C), neurons from FLRT2^+/−^ female mice presented with a significantly lower dendritic length; with a decreased number of ends and nodes (Fig. 5D–F), as well as with a lower number of dendritic intersections (Fig. 5G). Interestingly, when dendritic spines were examined, neurons from FLRT2^+/−^ female exhibited a significant decrease in number of thin spines, whereas Stubby, Mushroom and Long Thin spines appear unaltered (Fig. 5H).

## Discussion

While the identification of FLRT2 protein and its localization in the brain were described about eighteen years ago^19^, the functional roles of FLRT2 in the adult nervous system has remained virtually unknown. Data presented here derived from studies on FLRT2 haplodeficient (FLRT2^+/−^) mice points towards a potential functional involvement of FLRT2 in adult synaptic plasticity and memory recall and proposes FLRT2 as a molecular candidate implicated in the sex-dependent regulation of memory functions.

The use of FLRT2^+/−^ mice has precedents in the scientific literature, including studies addressing the functional relevance of FLRT2 in the morphogenesis of the developing heart^18^ and studies on the role of FLRT proteins in the developing brain reporting that FLRT2 proteins act as critical signaling molecules regulating cortical neuron migration and brain neuronal layering^22^. In these previous developmental studies^18,22^, the single allele deletion of the FLRT2 gene resulted in a significant reduction of FLRT2 protein levels. Here we extended the studies on FLRT2 to both sexes and to adulthood. Both female and male FLRT2^+/−^ adult mice examined here reached normal life spans, had no observable external physical defects, and presented with significant reduction in the levels of FLRT2 mRNA. Female and male FLRT2^+/−^ mice showed also regular brain morphologies, no detectable brain macroscopic cytoarchitectonical defects, and no prominent motoric dysfunctions. However, upon examination of hippocampus-dependent learning and memory using the Morris water maze, a significant augmentation in memory recall in female FLRT2^+/−^ mice was observed, whereas FLRT2^+/−^ male mice presented with a decrease in time spent in the target quadrant of the water tank. These observations suggest a bidirectional involvement of FLRT2 in hippocampal functions, possibly mediated by a sex-related signaling pathway. Our data showing sex-dependent differences in long-term potentiation during electrophysiological examinations *ex vivo*, and indicating an augmentation of synaptic plasticity only in female FLRT2^+/−^ mice, provide further support for this hypothesis.

Different brain neuronal LRR proteins have been implicated in adult neuroplasticity, neurogenesis, and memory storage^49–53^. In addition, the association between LRR-containing proteins and sex hormones activity in the brain^54,55^, as well as in other organs^56–58^, has been also previously described. Moreover, FLRT2 has been implicated in processes differentially regulated between males and females such as prostate cancer^59^ and chondrogenesis^60–62^ and in several primarily female and hormone-related physiological and pathophysiological processes in humans, including menarche^63^ and breast cancer^64,65^. In humans, different lines of evidence indicate that some of the differences in the cognitive function -and related pathologies- between males and females can be in part attributed to the differential regulation by sex-hormone signaling^66–72^. Studies in animal models, including mouse and rat, have also showed the importance of sex-related hormones and the sex-dependent differential effect of α- and β-estrogen receptors deficiency on neuronal morphology, synaptic plasticity, and memory functions^38–45^.

Interestingly, it has been recently shown that FLRT2 is differentially regulated by β-estradiol, the main female sex hormone^73^. Additionally, it has been also reported that estradiol deficiency demotes synapses and impairs long-term potentiation (LTP) exclusively in females^48,69^. Furthermore, male and female FLRT2^+/−^ mice studied here not only showed a sex-dependent reduction in α- and β-estrogen receptors, but only female FLRT2^+/−^ mice presented with reduced levels of the excitatory amino acid transporter 2 (EAAT2), thus suggesting a FLRT2-related synergistic crosstalk between sex-hormones and glutamatergic signaling. Further research is therefore required to verify and characterize this new molecular signaling path. EAAT2 (a.k.a. GLT-1), is a high affinity transporter for glutamate vastly expressed throughout the CNS that is accountable for more than 90% of glutamate reuptake in the CNS^74,75^. Additionally, increased levels of GLT-1 have been shown to exert negative effects on LTP and prolonged activation of the GLT-1 results in memory impairments in rats^76^. The regulation of GLT-1 by estradiol is very well established in non-neuronal brain cells^37,77,78^. Interestingly, it has been also shown that stimulation with β-estradiol results in an increased hippocampal GLT-1 expression^79^. These observations are in agreement with the improved MWM performance as well as with the enhancement of LTP and with the observed reduced protein levels of GLT-1 found here specifically in FLRT2^+/−^ females.

Here we also describe that whereas FLRT2 haplodeficiency results in augmented post-tetanic potentiation and enhanced memory recall in female brains, it also results in altered dendritic morphology, an observation at first glance rather paradoxical. One of the three members of the FLRT family of proteins, FLRT3 has shown similar effects *in vivo*. It has been additionally shown that a reduction in the levels of FLRT3 (which has a completely different pattern of tissue localization compared to FLRT2) reduces afferent input strength and dendritic spine number in neurons from the dentate gyrus^21^. During development, both FLRT2 and FLRT3 proteins have been shown to have dual -and rather opposing- roles as neuronal adhesion and repulsion molecular elements, although the pathways underlying these actions have only recently begun to be identified and characterized^22,80^. FLRT proteins are also known to modulate Growth Factor signaling^16,17,81,82^ and our data here further suggest a potential implication of sex-hormone dependent mechanisms of regulation of the FLRT2 function. Thus, how FLRT2 can concomitantly enhance plasticity-mediated responses and decrease apical dendritic length and thin spine numbers remains to be elucidated. One could however, cautiously speculate that FLRT2 could -for example- mediate in the promotion of dendritic spine clustering, which is known to result in the nonlinear summation of synaptic inputs thus inducing synaptic strengthening^83^. FLRT2 could also dually influence spine turnover or negatively regulate the number of basal spines versus positively boosting the activity-dependent increase in the number of entirely newly formed spines during plastic events (see also^47^). The growing variety of FLRT2 signal complexity makes also plausible to propose that perhaps the here observed, transitory enhancement of plasticity and memory recall is concomitant with alterations in dendritic complexity in female FLRT2^+/−^ neurons due to the induction of a gene-dependent compensatory mechanism (see also^84,85^). As also known for other proteins^86^, one example of this compensation could in fact be the here reported decrease in the levels of the EAAT2 transporter, which could contribute to increase the levels of excitatory neurotransmitter in the synaptic cleft so all dendritic spines would be receiving stronger synaptic inputs. Additional research is therefore required to clarify the diversity of FLRT2 functions in the adult brain and to explore the possible existence of a molecular feedback mechanism engaged in homeostatically regulating the neuronal effects of FLRT2 down-regulation in a sex-dependent manner.

Taken together, our data propose FLRT2 as a novel molecular element that might importantly contribute *in vivo* to learning-related synaptic plasticity in the adulthood, a hypothesis that also invites for further research in the field of the sexually dysmorphic cognitive function and FLRT proteins in both physiological and pathophysiological contexts.

## Materials and Methods

### Animals

FLRT2^*+/*−^ animals were a generous gift from Prof. Elizabeth Robertson (Sir William Dunn School of Pathology, University of Oxford)^18^. FLRT2^*+/*−^ mice were bred and maintained in specific pathogen-free facilities of the Medical University of Vienna and used at 8–20 weeks for behavioral and electrophysiological experiments. Animals were housed in groups of 3–5 mice in a temperature ((22 ± 1) °C) and light ((200 ± 20) lx) controlled colony room; food and water were provided *ad libitum*. The room was kept on a 12 h light/dark cycle with light period starting at 6:00 a.m. Experiments described in this study were approved by the Bundesministerium für Wissenschaft, Forschung und Wirtschaft (GZ-66.009/0158-WF/V/3b/2016), carried out according to EU-directive 2010/63/EU and are in compliance with ARRIVE guidelines.

### qRT-PCR

Animals were sacrificed by neck-dislocation and brain dissection performed in ice-cold aCSF solution. Following hippocampi collection, tissue was stored at -20 °C in RNA later® solution (Ambion, Austin, Texas). qRT-PCR was performed as described elsewhere with minor modifications^87^. Briefly, RNA isolation was performed using the Extractme Total RNA Kit (Cat. No. EM09-100, DNA-Gdansk, Gdansk, Poland) following manufacturer recommendations. Next, using the RevertAid First Strand cDNA Synthesis Kit (Cat. No. K1621, Thermo Scientific, Vienna, Austria), 1000 ng of total RNA was used per reaction to synthesize cDNA in accordance with manufacturer’s instructions. For PCR amplification, 1:5 dilution of cDNA was mixed with the GoTaq qPCR Master Mix (Cat. No. A6002, Promega Corporation, Madison, WI, USA) and amplified on a StepOnePlus real-time PCR system (serial no. 271000455; Applied Biosystems, Foster City, CA). All reactions were prepared in triplicates. The protocol used comprised of 40 cycles, with four steps (95 °C, 5 min; 95 °C, 30 s; 60 °C, 60 s; 72 °C, 30 s). Primers used had following sequences: FLRT2 FW, 5′-GATTCGGATGAGCTAAGGAG; RV, 5′-TTTGAGGATGAAAGCCCCAC^18^; Estrogen receptor alpha (ERα) FW: ATGAA AGGCG GCATA CGGAA AG, RV: CACCC ATTTC ATTTC GGCCT TC; Estrogen receptor beta (ERβ) FW: CCAGA CTGCA AGCCC AAATG T, RV: AGAAG CGATG ATTGG CAGTG G. For data analysis Livak method was used^88^ with β-Actin (FW: ATGGTGGGAATGGGTCAGAAG, RV: TCTCCATGTCGTCCCAGTTG) as a reference gene.

### Behavioral testing

All experiments conducted in this study were carried out during the light phase of the animals day-night cycle by an experimenter blinded to the genotype of the animals. Animal numbers are specified in figure legends. At least one week before the behavioral testing, mice were single caged and handled for 5 min daily in order to reduce stress from manipulations throughout the experiments. Additionally, at least 1 h before each experiment mice were placed in the room where behavioral testing was performed for habituation.

### Open field test

Mice were placed in the center of an Open Field (ENV-510, Med. Associates Inc., St. Albans, VT) consisting of 4 transparent walls (height 20.3 cm) made of acrylic glass mounted on a white polyvinyl chloride plastic board, and allowed to explore the arena for 40 min. The surface area of the arena was 27.3 cm × 27.3 cm, with light intensity of 300 lx in the center. Average velocity and the distance traveled were recorded and analyzed using Activity Monitor software (SOF-812, Med. Associates Inc., St. Albans, VT). To test for any possible locomotor deficits, average velocity and distance traveled during the test were analyzed and compared to the wild type.

### Rota-rod

The Rota-Rod test was performed as previously described^89–91^. Briefly, each animal had a 60 s accommodation period (pre-training) before starting with the first trial on the Rota-rod Treadmill (USB Rota Rod “SOF-ENV-57X”, MedAssociates Inc., St. Albans USA). The speed at the start was 4 rpm and was gradually accelerated until 40 rpm during the period of 5 min. The trial was stopped either at the end of 5 min or when the animal falls of the rod. The time spent on the Rota-rod was automatically recorded and used to evaluate the performance of mice. Each animal’s performance was recorded in 3 trials with 15 min breaks in-between and the average was used to check for any deficits in motor coordination in FLRT2^+/−^ mice as compared to their wild type littermates^92^.

### Morris Water Maze

We used published experimental setups and protocols with minor modifications^89–91^. The tank (122 cm diameter, 76 cm height) was filled with tap water at room temperature ((22 ± 1) °C, 48 cm depth). Water was made opaque with non-toxic white paint (Viewpoint, France). Motor adeptness and visual capacity of mice were checked on one pre-training day (two trials). During visual acuity examination, a visible square platform (10 cm) with a red flag was placed in the tank. If the animal was not able to locate the platform within 2 min, it was guided to it. On the first training day, the platform was submerged 1.5 cm beneath the water surface. Once placed in the pool of water, animals had to find the hidden platform. Handmade distal cues were mounted outside of the pool as navigation aids. Following protocols previously used with success by our group^89,91^, each mouse was given 3 trials per day for 3 training days (TD) with inter-trial period of 1 h. Latencies to reach the platform and swimming speeds were recorded/analyzed using an automated system (Monochrome/Near Infra-red camera coupled to tracking software: Videotrack [02-WATERMAZE-NSHD-LBW], Viewpoint, France). A probe trial (PT) was conducted 24 h after the last training trial. For this, the platform was removed to test memory recall and animals were allowed to swim for 30 s. Times spent in the target quadrant (in which platform used to be) were used as a readout of long-term memory.

### Electrophysiology

#### Hippocampal slices preparation

Acute hippocampal slices were prepared as previously described^91,93,94^, with minor modifications. Following isoflurane-induced anesthesia, animals were decapitated and brains extracted into a cold artificial cerebrospinal fluid (aCSF) solution containing (in mM): 125 NaCl, 2.5 KCl, 25 NaHCO_3_, 2 CaCl_2_, 1 MgCl_2_, 25 D-glucose, 1.25 NaH_2_PO_4_ with pH adjusted to 7.4. Transverse hippocampal slices (400 μm thick) were prepared using a McIlwain Tissue Chopper (Mickle Laboratory Engineering, Guildford, Surrey, UK) and transferred to the homemade recovery chamber at (28 ± 2) °C for 1 h. All solutions were constantly supplied with carbogen gas mixture (95% O_2_, 5% CO_2_).

### Extracellular recordings

To record evoked field excitatory postsynaptic potentials (fEPSPs) borosilicate glass pipettes (2–5 MΩ) were filled with aCSF solution and used as recording electrodes, located ~400 μm from the Teflon-coated tungsten wire bipolar stimulating electrode (~50 µm diameter tip) at the CA3-Schaffer Collateral-CA1 synaptic region, as previously published^91^. Input/Output (I/O) curves were obtained by repeated stimulation with pulses of voltage (0–6 V, 1 V increments, 100 μs duration) with an inter-stimulus interval of 10 s. The decaying slopes of field potentials were normalized to the maximum value and the average was compared between FLRT2^+/−^ and wild type mice of both sexes. In long-term potentiation (LTP), stimulation inducing 40–50% of maximal amplitudes were used for baseline (15 min) recordings (pulse duration = 100 μs, 0.03 Hz). LTP was induced by 5 trains/100 Hz (10 μs/pulse, 4 s interval between trains, same amplitude/duration as for base line recordings). Synaptic potentiation was determined by analyzing the changes in the slopes of the fEPSP decaying phase using baseline values as reference. AxoClamp-2B amplifier (Bridge mode) and a Digidata-1440 interface (Axon Instruments, MolecularDevices, Berkshire, UK) were used for recordings and pClamp-10 software (Molecular Devices,Berkshire, UK) was used for offline analysis.

### Dendritic Morphology Analysis

Brains from FLRT^+/+^ (n = 5) and FLRT^+/−^ (n = 4) mice (8 weeks old) were used for neuronal morphological analysis with the FD Rapid GolgiStainTM kit following the instructions of the manufacturer (Cat Nr. PK401, FD Neurotechnologies, INC). Blind analysis was performed on tertiary branches of the apical trees of pyramidal neurons from the CA1 hippocampal region, an area widely known for its implication in learning and memory^95,96^ and where FLRT2 proteins are endogenously expressed^22,23^. Randomly picked, consistently impregnated neurons relatively isolated from adjacent impregnated neurons were selected to ensure that the examined dendrites originated from identified cells. The morphometric properties of neurons were examined using the Neurolucida (version 10) system. At least four neurons per brain (two per brain hemisphere) were used for analysis. For dendritic spine analysis, a segment of 40 µm per neuron was examined. Spine type was determined attending to the following morphometric parameters (length/width < 1 = Stubby; length/width < 1 and width > 0.6 µm = Mushroom; length/width > 1 and length < 1 µm = Thin; length/width > 1 and 1 µm ≤ length < 2 µm = Long Thin).

### Western blot

Total membrane proteins were isolated as previously described^97^. Hippocampi were homogenized in Synaptic Protein Extraction Reagent (Syn-PER, Thermo Fisher Scientific, MA USA) and centrifuged in a bench top centrifuge at 1200 × g for 10 min at 4 °C. In addition, supernatants were collected and centrifuged at 15,000 × g for 20 min at 4 °C. Pellets with the synaptosomal fraction were solubilized in a buffer containing: 1.5% SDS, 100 mM NaCl, 20 mM Tris–HCl pH 7.5, Protease Inhibitor Cocktail (Sigma Aldrich, Vienna, Austria), and gently agitated at room temperature for 1 h. Next, protein concentrations of samples were determined using the BCA protein assay kit (Pierce, Rockford, USA). Following the protein quantification, the samples were mixed with 4 × Laemmli Sample Buffer (Bio-Rad Laboratories, Richmond, CA, USA) and incubated for 30 min at room temperature. Electrophoresis was performed using the Criterion electrophoresis cell (Bio-Rad Laboratories, Richmond, CA, USA) with 10 µg of protein per sample loaded on 1D-SDS-PAGE (10% separating gel), and followed by the transfer of proteins onto the PVDF membrane (Millipore, Darmstadt, Germany) at 350 mA of constant current for 2 h at 6 °C using a semi-dry transfer system (PerfectBlue™ Electroblotter Sedec™ M, Peqlab Biotechnologie GmbH, Erlagen, Germany). Next, membranes were blocked in TBS containing 5% non-fat dry milk for 1 h at room temperature, followed by overnight incubation with diluted primary antibody at 4 °C. Antibodies used were VGLUT1 (1:1000, gunea pig, Cat. No. MAB5502, Merck Millipore), EAAT2/GLT-1 (1:1000, rabbit, Cat.No. 41621, Abcam) and β-Actin (1:2000, Cat. No. A0760-40A, US Biological). On the following day, membranes were washed 3 × by gentle agitation in TBS containing 0.1% Tween-20 and were incubated with peroxidase-coupled anti-rabbit IgG (1:2000; Cat. No. 7074, Cell Signaling,), anti-mouse IgG (1:2000, Cat. No. 7076, Cell Signaling) and anti-guinea pig IgG (1:10000; Cat. No. 706-035-148, Jackson Immuno Research) secondary antibody for 1 h at room temperature. Membranes were developed with ECL Western blotting detection system (Bio-Rad Laboratories) and proteins visualized by FluorChem HD2 system (Alpha Innotech, San Leandro, CA, USA). Protein levels were quantified by densitometry using ImageJ software (http://rsbweb.nih.gov/ij/).

### Statistical analysis

Statistical analyses were carried out using GraphPad Prism Software, version 7.0 (San Diego, CA, USA). T-test was used for comparisons between two groups and two-way ANOVA followed by Tukey’s multiple comparisons tests as *posthoc* comparisons for multiple groups where appropriate. For latency, swimming speed, Input/output curves and long-term potentiation measurements two-way repeated measures ANOVA was used. P values ≤ 0.05 were considered significant. All data are expressed as means +/− standard error.

## Electronic supplementary material

Supplementary Data

## References

[1] Kobe B, Deisenhofer J (1995). Proteins with leucine-rich repeats. Curr Opin Struct Biol.

[2] Kobe B, Deisenhofer J (1995). A structural basis of the interactions between leucine-rich repeats and protein ligands. Nature.

[3] Kobe B, Kajava AV (2001). The leucine-rich repeat as a protein recognition motif. Curr Opin Struct Biol.

[4] Levine ES, Dreyfus CF, Black IB, Plummer MR (1995). Brain-derived neurotrophic factor rapidly enhances synaptic transmission in hippocampal neurons via postsynaptic tyrosine kinase receptors. Proceedings of the National Academy of Sciences of the United States of America.

[5] Xu B (2000). The role of brain-derived neurotrophic factor receptors in the mature hippocampus: modulation of long-term potentiation through a presynaptic mechanism involving TrkB. J Neurosci.

[6] Edwards TL (2009). An association analysis of Alzheimer disease candidate genes detects an ancestral risk haplotype clade in ACE and putative multilocus association between ACE, A2M, and LRRTM3. Am J Med Genet B Neuropsychiatr Genet.

[7] Majercak J (2006). LRRTM3 promotes processing of amyloid-precursor protein by BACE1 and is a positional candidate gene for late-onset Alzheimer’s disease. Proceedings of the National Academy of Sciences of the United States of America.

[8] Watanabe T, von der Kammer H, Wang X, Shintani Y, Horiguchi T (2012). Neuronal expression of F-box and leucine-rich-repeat protein 2 decreases over Braak stages in the brains of Alzheimer’s disease patients. Neuro-degenerative diseases.

[9] Inoue H (2007). Inhibition of the leucine-rich repeat protein LINGO-1 enhances survival, structure, and function of dopaminergic neurons in Parkinson’s disease models. Proceedings of the National Academy of Sciences of the United States of America.

[10] MacLeod D (2006). The familial Parkinsonism gene LRRK2 regulates neurite process morphology. Neuron.

[11] Schapansky J, Nardozzi JD, LaVoie MJ (2015). The complex relationships between microglia, alpha-synuclein, and LRRK2 in Parkinson’s disease. Neuroscience.

[12] Zimprich A (2004). Mutations in LRRK2 cause autosomal-dominant parkinsonism with pleomorphic pathology. Neuron.

[13] Gu W (2002). The LGI1 gene involved in lateral temporal lobe epilepsy belongs to a new subfamily of leucine-rich repeat proteins. FEBS letters.

[14] Kalachikov S (2002). Mutations in LGI1 cause autosomal-dominant partial epilepsy with auditory features. Nat Genet.

[15] Smith TG, Tickle C (2006). The expression of Flrt3 during chick limb development. The International journal of developmental biology.

[16] Bottcher RT, Pollet N, Delius H, Niehrs C (2004). The transmembrane protein XFLRT3 forms a complex with FGF receptors and promotes FGF signalling. Nature cell biology.

[17] Haines BP, Wheldon LM, Summerbell D, Heath JK, Rigby PW (2006). Regulated expression of FLRT genes implies a functional role in the regulation of FGF signalling during mouse development. Developmental biology.

[18] Muller PS (2011). The fibronectin leucine-rich repeat transmembrane protein Flrt2 is required in the epicardium to promote heart morphogenesis. Development (Cambridge, England).

[19] Lacy SE, Bonnemann CG, Buzney EA, Kunkel LM (1999). Identification of FLRT1, FLRT2, and FLRT3: a novel family of transmembrane leucine-rich repeat proteins. Genomics.

[20] Tsuji L (2004). FLRT3, a cell surface molecule containing LRR repeats and a FNIII domain, promotes neurite outgrowth. Biochemical and biophysical research communications.

[21] O’Sullivan ML (2012). FLRT proteins are endogenous latrophilin ligands and regulate excitatory synapse development. Neuron.

[22] Yamagishi S (2011). FLRT2 and FLRT3 act as repulsive guidance cues for Unc5-positive neurons. The EMBO journal.

[23] de Wit J, Ghosh A (2014). Control of neural circuit formation by leucine-rich repeat proteins. Trends Neurosci.

[24] Geist PA, Dulka BN, Barnes A, Totty M, Datta S (2017). BNDF heterozygosity is associated with memory deficits and alterations in cortical and hippocampal EEG power. Behavioural brain research.

[25] Chen KS (1997). Disruption of a single allele of the nerve growth factor gene results in atrophy of basal forebrain cholinergic neurons and memory deficits. J Neurosci.

[26] Wang Y, Cheng A, Mattson MP (2006). The PTEN phosphatase is essential for long-term depression of hippocampal synapses. Neuromolecular Med.

[27] Egea J (2008). *Genetic ablation of FLR*T3 reveals a novel morphogenetic function for the anterior visceral endoderm in suppressing mesoderm differentiation. Genes & development.

[28] Leyva-Diaz E (2014). FLRT3 is a Robo1-interacting protein that determines Netrin-1 attraction in developing axons. Curr Biol.

[29] Tai-Nagara I (2017). Placental labyrinth formation in mice requires endothelial FLRT2/UNC5B signaling. Development (Cambridge, England).

[30] Maretto S (2008). Ventral closure, headfold fusion and definitive endoderm migration defects in mouse embryos lacking the fibronectin leucine-rich transmembrane protein FLRT3. Developmental biology.

[31] Kovacs AD, Pearce DA (2013). Location- and sex-specific differences in weight and motor coordination in two commonly used mouse strains. Sci Rep.

[32] Vorhees CV, Williams MT (2006). Morris water maze: procedures for assessing spatial and related forms of learning and memory. Nature protocols.

[33] Wolf A, Bauer B, Abner EL, Ashkenazy-Frolinger T, Hartz AM (2016). A Comprehensive Behavioral Test Battery to Assess Learning and Memory in 129S6/Tg2576 Mice. PloS one.

[34] Wilson MA, Tonegawa S (1997). Synaptic plasticity, place cells and spatial memory: study with second generation knockouts. Trends Neurosci.

[35] Balschun D (2010). Vesicular glutamate transporter VGLUT1 has a role in hippocampal long-term potentiation and spatial reversal learning. Cereb Cortex.

[36] Karki P (2014). Mechanism of raloxifene-induced upregulation of glutamate transporters in rat primary astrocytes. Glia.

[37] Pawlak J, Brito V, Kuppers E, Beyer C (2005). Regulation of glutamate transporter GLAST and GLT-1 expression in astrocytes by estrogen. Brain Res Mol Brain Res.

[38] Liu F (2008). Activation of estrogen receptor-beta regulates hippocampal synaptic plasticity and improves memory. Nature neuroscience.

[39] Sanchez-Andrade G, Kendrick KM (2011). Roles of alpha- and beta-estrogen receptors in mouse social recognition memory: effects of gender and the estrous cycle. Hormones and behavior.

[40] Gupta RR, Sen S, Diepenhorst LL, Rudick CN, Maren S (2001). Estrogen modulates sexually dimorphic contextual fear conditioning and hippocampal long-term potentiation (LTP) in rats(1). Brain Res.

[41] Hadjimarkou, M. M. & Vasudevan, N. GPER1/GPR30 in the brain: Crosstalk with classical estrogen receptors and implications for behavior. *J Steroid Biochem Mol Biol* (2017). 10.1016/j.jsbmb.2017.04.012 28465157

[42] Hart D (2014). Activation of the G-protein coupled receptor 30 (GPR30) has different effects on anxiety in male and female mice. Steroids.

[43] Murakami G (2015). Estrogen receptor KO mice study on rapid modulation of spines and long-term depression in the hippocampus. Brain Res.

[44] Xiong YS (2015). Opposite effects of two estrogen receptors on tau phosphorylation through disparate effects on the miR-218/PTPA pathway. Aging cell.

[45] Zuloaga DG, Zuloaga KL, Hinds LR, Carbone DL, Handa RJ (2014). Estrogen receptor beta expression in the mouse forebrain: age and sex differences. J Comp Neurol.

[46] Woolley CS, Weiland NG, McEwen BS, Schwartzkroin PA (1997). Estradiol increases the sensitivity of hippocampal CA1 pyramidal cells to NMDA receptor-mediated synaptic input: correlation with dendritic spine density. J Neurosci.

[47] Engert F, Bonhoeffer T (1999). Dendritic spine changes associated with hippocampal long-term synaptic plasticity. Nature.

[48] Vierk R, Brandt N, Rune GM (2014). Hippocampal estradiol synthesis and its significance for hippocampal synaptic stability in male and female animals. Neuroscience.

[49] Okun E (2010). Toll-like receptor 3 inhibits memory retention and constrains adult hippocampal neurogenesis. Proceedings of the National Academy of Sciences of the United States of America.

[50] Rolls A (2007). Toll-like receptors modulate adult hippocampal neurogenesis. Nature cell biology.

[51] Chao MV (2003). Neurotrophins and their receptors: a convergence point for many signalling pathways. Nature reviews.

[52] Minichiello L (2002). Mechanism of TrkB-mediated hippocampal long-term potentiation. Neuron.

[53] Kang H, Welcher AA, Shelton D, Schuman EM (1997). Neurotrophins and time: different roles for TrkB signaling in hippocampal long-term potentiation. Neuron.

[54] Wang W (2016). Estrogen’s Effects on Excitatory Synaptic Transmission Entail Integrin and TrkB Transactivation and Depend Upon beta1-integrin function. Neuropsychopharmacology.

[55] Thakkar R (2016). NLRP3 Inflammasome Activation in the Brain after Global Cerebral Ischemia and Regulation by 17beta-Estradiol. Oxid Med Cell Longev.

[56] Charpentier AH (2000). Effects of estrogen on global gene expression: identification of novel targets of estrogen action. Cancer research.

[57] Koizumi M (2015). Lgr4 controls specialization of female gonads in mice. Biol Reprod.

[58] Sun X (2014). Ovarian LGR5 is critical for successful pregnancy. FASEB J.

[59] Wu Y (2016). Methylation profiling identified novel differentially methylated markers including OPCML and FLRT2 in prostate cancer. Epigenetics.

[60] Flintoff KA, Arudchelvan Y, Gong SG (2014). FLRT2 interacts with fibronectin in the ATDC5 chondroprogenitor cells. J Cell Physiol.

[61] Gong SG, Mai S, Chung K, Wei K (2009). Flrt2 and Flrt3 have overlapping and non-overlapping expression during craniofacial development. Gene Expr Patterns.

[62] Xu Y, Wei K, Kulyk W, Gong SG (2011). FLRT2 promotes cellular proliferation and inhibits cell adhesion during chondrogenesis. J Cell Biochem.

[63] Demerath EW (2013). Genome-wide association study of age at menarche in African-American women. Hum Mol Genet.

[64] Ordway JM (2007). Identification of novel high-frequency DNA methylation changes in breast cancer. PloS one.

[65] Bae H (2017). Epigenetically regulated Fibronectin leucine rich transmembrane protein 2 (FLRT2) shows tumor suppressor activity in breast cancer cells. Sci Rep.

[66] Arevalo MA, Azcoitia I, Gonzalez-Burgos I, Garcia-Segura LM (2015). Signaling mechanisms mediating the regulation of synaptic plasticity and memory by estradiol. Hormones and behavior.

[67] Boulware MI, Mermelstein PG (2005). The influence of estradiol on nervous system function. Drug News Perspect.

[68] Brinton RD (2008). Progesterone receptors: form and function in brain. Front Neuroendocrinol.

[69] Fester L, Rune GM (2015). Sexual neurosteroids and synaptic plasticity in the hippocampus. Brain Res.

[70] McCarthy MM (2009). The epigenetics of sex differences in the brain. J Neurosci.

[71] Mukai H (2010). Modulation of synaptic plasticity by brain estrogen in the hippocampus. Biochimica et biophysica acta.

[72] Zhao L, Woody SK, Chhibber A (2015). Estrogen receptor beta in Alzheimer’s disease: From mechanisms to therapeutics. Ageing Res Rev.

[73] Coughlan N, Thillainadesan G, Andrews J, Isovic M, Torchia J (2013). beta-Estradiol-dependent activation of the JAK/STAT pathway requires p/CIP and CARM1. Biochimica et biophysica acta.

[74] Rao VH (2015). Erbb2 up-regulation of ADAM12 expression accelerates skin cancer progression. Molecular carcinogenesis.

[75] Beart PM, O’Shea RD (2007). Transporters for L-glutamate: an update on their molecular pharmacology and pathological involvement. British journal of pharmacology.

[76] Matos-Ocasio F, Hernandez-Lopez A, Thompson KJ (2014). Ceftriaxone, a GLT-1 transporter activator, disrupts hippocampal learning in rats. Pharmacology, biochemistry, and behavior.

[77] Lee E, Sidoryk-Wegrzynowicz M, Farina M, Rocha JB, Aschner M (2013). Estrogen attenuates manganese-induced glutamate transporter impairment in rat primary astrocytes. Neurotox Res.

[78] Lee E (2012). Transforming growth factor-alpha mediates estrogen-induced upregulation of glutamate transporter GLT-1 in rat primary astrocytes. Glia.

[79] Sarfi M, Elahdadi Salmani M, Goudarzi I, Lashkar Boluki T, Abrari K (2017). Evaluating the role of astrocytes on beta-estradiol effect on seizures of Pilocarpine epileptic model. European journal of pharmacology.

[80] Seiradake E (2014). FLRT structure: balancing repulsion and cell adhesion in cortical and vascular development. Neuron.

[81] Wei K, Xu Y, Tse H, Manolson MF, Gong SG (2011). Mouse FLRT2 interacts with the extracellular and intracellular regions of FGFR2. Journal of dental research.

[82] Wheldon LM (2010). Critical role of FLRT1 phosphorylation in the interdependent regulation of FLRT1 function and FGF receptor signalling. PloS one.

[83] Pereira AC (2014). Glutamatergic regulation prevents hippocampal-dependent age-related cognitive decline through dendritic spine clustering. Proceedings of the National Academy of Sciences of the United States of America.

[84] Poo MM (2016). What is memory? The present state of the engram. BMC Biol.

[85] Rabinowitch I, Segev I (2008). Two opposing plasticity mechanisms pulling a single synapse. Trends Neurosci.

[86] Kim S (2015). Network compensation of cyclic GMP-dependent protein kinase II knockout in the hippocampus by Ca2 + -permeable AMPA receptors. Proceedings of the National Academy of Sciences of the United States of America.

[87] Schaufler J (2016). Fluoxetine normalizes disrupted light-induced entrainment, fragmented ultradian rhythms and altered hippocampal clock gene expression in an animal model of high trait anxiety- and depression-related behavior. Annals of medicine.

[88] Livak KJ, Schmittgen TD (2001). Analysis of relative gene expression data using real-time quantitative PCR and the 2(-Delta Delta C(T)) Method. Methods.

[89] Khan D (2014). Long-term effects of maternal immune activation on depression-like behavior in the mouse. Translational psychiatry.

[90] Kim, E. J. *et al*. Alzheimer’s disease risk factor lymphocyte-specific protein tyrosine kinase regulates long-term synaptic strengthening, spatial learning and memory. *Cell Mol Life Sci* (2012). 10.1007/s00018-012-1168-1 PMC11113176 23007847

[91] Cicvaric, A. *et al*. The brain-tumor related protein podoplanin regulates synaptic plasticity and hippocampus-dependent learning and memory. *Annals of medicine*, 1–17 (2016). 10.1080/07853890.2016.1219455 PMC5125287 27558977

[92] Rogers J, Maurizio SJ (1999). More than just ‘skin deep’. Rdh.

[93] Monje FJ (2012). Proteomics reveals selective regulation of proteins in response to memory-related serotonin stimulation in Aplysia californica ganglia. Proteomics.

[94] Rammes G (2009). Isoflurane anaesthesia reversibly improves cognitive function and long-term potentiation (LTP) via an up-regulation in NMDA receptor 2B subunit expression. Neuropharmacology.

[95] Van Hoesen GW, Hyman BT (1990). Hippocampal formation: anatomy and the patterns of pathology in Alzheimer’s disease. Prog Brain Res.

[96] Nguyen PV, Abel T, Kandel ER (1994). Requirement of a critical period of transcription for induction of a late phase of LTP. Science (New York, N.Y.

[97] Bai F, Witzmann FA (2007). Synaptosome proteomics. Subcell Biochem.

