# Supplementary material for: Enhanced synaptic plasticity and spatial memory in female but not male FLRT2-haplodeficient mice
Source: Sci Rep. 2018 Feb 27;8:3703. doi: 10.1038/s41598-018-22030-4 (PMC5829229; doi:10.1038/s41598-018-22030-4)

Enhanced synaptic plasticity and spatial memory in female but not male FLRT2-haplodeficient mice

Ana Cicvaric[1#], Jiaye Yang[1#], Tanja Bulat[1], Alice Zambon[1], Manuel Dominguez-Rodriguez[1], Rebekka Kühn[1], Michael G. Sadowicz[1], Anjana Siwert[1], Joaquim Egea[2], Daniela D. Pollak[1], Thomas Moeslinger[3] and Francisco J. Monje[1]*

[1] *Department of Neurophysiology and Neuropharmacology, Center for Physiology and Pharmacology, Medical University of Vienna, Schwarzspanierstrasse 17, 1090 Vienna, Austria.*

[2] *Molecular and Developmental Neurobiology Research Group, Universitat de Lleida - IRBLleida, Office 1.13, Lab. 1.06. Avda. Rovira Roure, 80, 25198, Lleida, Spain.*

[3] *Institute for Physiology, Center for Physiology and Pharmacology, Medical University of Vienna, Schwarzspanierstrasse 17, 1090 Vienna, Austria.*

[*]Correspondence:

Assoc. Prof. Dr. Francisco J. Monje

Department of Neurophysiology & Neuropharmacology

Center for Physiology and Pharmacology, Medical University of Vienna.

Schwarzspanierstrasse 17

A-1090 Vienna, Austria

francisco.monje@meduniwien.ac.at

Supplementary Data

Supplementary Figure 1. FLRT2 haplodeficiency and hippocampal levels of VGlut1 and EAAT2. Original pictures of full-length membranes used in Western blot assays and densitometric analyses as described in Figure 4 of the main manuscript. A) Membrane incubated with the EAAT2 antibody (a.k.a., GLT-1; Cat.No. 41621, Abcam) and with a β-Actin antibody (Cat. No. A0760-40A, US Biological). NMDAR2B was not used in this study. B) Membrane incubated with the VGlut1 antibody (Cat. No. MAB5502, Merck Millipore) and re-incubated with a β-Actin antibody (Cat. No. A0760-40A, US Biological). Arrowed lane not used for analysis.

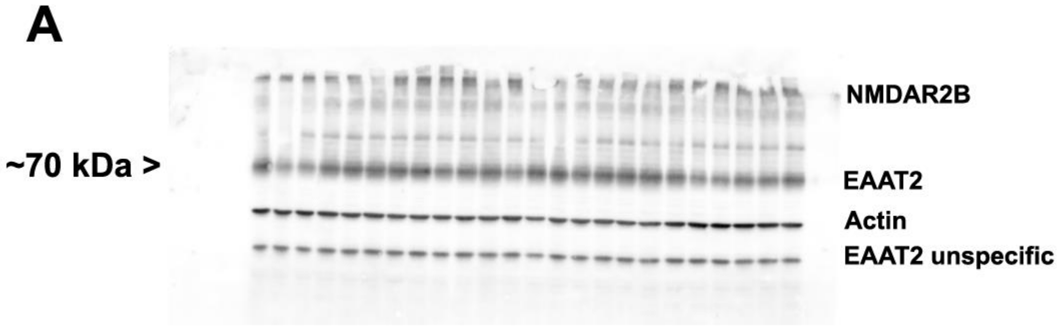

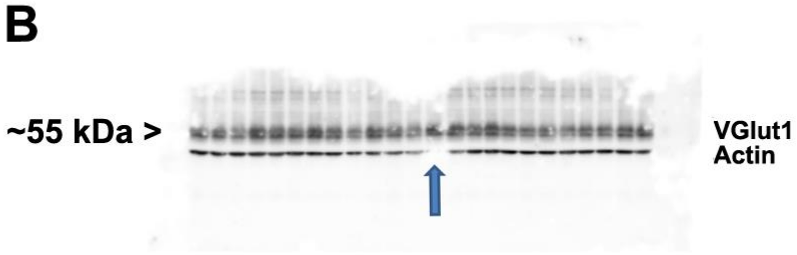

Supplement: Supplementary file 1 — Supplementary Data [file 41598_2018_22030_MOESM1_ESM.pdf]
